# Identification of Differentially Expressed lncRNAs in Response to Blue Light and Expression Pattern Analysis of *Populus tomentosa* Hybrid Poplar *741*

**DOI:** 10.3390/plants12173157

**Published:** 2023-09-02

**Authors:** Hongyan Li, Yiwen Zhang, Jinping Lan, Shijie Wang, Hongyu Cai, Xin Meng, Yachao Ren, Minsheng Yang

**Affiliations:** 1 Forest Department, Forestry College, Hebei Agricultural University, Baoding 071000, China; 18732377741@163.com (H.L.); 13930298724@163.com (Y.Z.); wangshijieem@126.com (S.W.); caihy0817@163.com (H.C.); mxailhy@126.com (X.M.); renyachao@hebau.edu.cn (Y.R.); 2 Hebei Key Laboratory for Tree Genetic Resources and Forest Protection, Baoding 071000, China; 3 Life Science Research Center, Hebei North University, Zhangjiakou 075000, China; lanjimping_mbb@126.com

**Keywords:** light response, *Populus 741*, lncRNAs, ceRNAs, growth and development

## Abstract

Poplar is an important shelterbelt, timber stand, and city tree species that has been the focus of forestry research. The regulatory role of the long non-coding RNA molecule (lncRNA; length > 200 nt) has been a research hotspot in plants. In this study, seedlings of *741* poplar were irradiated with LED blue and white light, and the Illumina HiSeq 2000 sequencing platform was used to identify lncRNAs. |logFC| > 1 and *p* < 0.05 were considered to indicate differentially expressed lncRNAs, and nine differentially expressed lncRNAs were screened, the target genes of which were predicted, and three functionally annotated target genes were obtained. The differentially expressed lncRNAs were identified as miRNA targets. Six lncRNAs were determined to be target sites for twelve mRNAs in six miRNA families. LncRNAs and their target genes, including lncRNA *MSTRG.20413.1-ptc-miR396e-5p-GRF9*, were verified using quantitative real-time polymerase chain reaction analysis, and the expression patterns were analyzed. The analysis showed that the *ptc-miR396e-5p* expression was downregulated, while lncRNA *MSTRG.20413.1* and *GRF9* expression was upregulated, after blue light exposure. These results indicate that lncRNAs interact with miRNAs to regulate gene expression and affect plant growth and development.

## 1. Introduction

Poplar (dicotyledon, Salicaceae, *Populus*) is a perennial deciduous tree species widely cultivated in China and an important shelterbelt, timber stand, and city tree species. It has been used to improve ecological structures and provides photosynthates that purify the air [1]. Due to its rapid growth and high productivity, poplar has become a model for studying woody plants [2]. As the energy source of photosynthesis, light is an important environmental factor affecting plant growth and development, and its quality has an important effect on plant physiology and morphogenesis, as well as in regulating plant growth [3,4,5]. Many photoreceptors exist in the plant body that are involved in light irradiation-induced photoresponses. It has been proved that red and blue light affect plant growth and development more than other spectral regions [6]. In recent years, scholars have been interested in how blue light affects plant growth. He et al. irradiated tomatoes grown in greenhouses with blue light and found that different frequencies of blue light treatments significantly increased the amounts of lycopene, total phenolics, total flavonoids, vitamin C, and soluble sugar in tomato fruits. Blue light treatment also promotes ethylene release and accelerated fruit maturation [7]. Ren et al. showed that the combined application of red and blue light can regulate the expression of photosynthesis-related genes in rice leaves, affecting the activity of the Rubisco enzyme, and consequently influencing rice photosynthesis [8]. The effect of light on plants varies by species, and there have been few reports on the response of woody plants to blue light. Therefore, this study analyzed the response of poplar *741* to blue light and investigated the specific molecular mechanisms.

During various biological processes, long-noncoding RNAs (lncRNAs) play a key role at the level of gene expression [9]. LncRNAs are mainly RNA molecules with lengths > 200 bp and no coding ability, produced by RNA polymerase II transcription. They have a 5′ cap structure and 3′ polyA tail. Their expression level is lower than that of mRNAs [10,11]. LncRNAs commonly lack conserved sequences between species and are often differentially expressed among different tissues and cells [12,13]. High-throughput sequencing has identified a great deal of lncRNAs in various plants, such as *Oryza sativa* L. [14], *Zea mays* L. [15], *Triticum aestivum* L. [16], *Gossypium hirsutum* L. [17], and *Brassica* [18]. LncRNAs can be divided into functional categories, i.e., signaling, bait, guide, and scaffold molecules [19]. The most well-studied mechanism of lncRNA action is the competing endogenous RNAs (ceRNA) bait mechanism; some lncRNAs and protein-coding mRNAs in cells are highly homologous in sequence, resulting in an miRNA binding competition between lncRNAs and mRNAs [20].

It has previously been found that lncRNAs participate in a variety of plant growth and developmental processes, as well as responses to biological and abiotic stressors. The sequencing of *Populus* × *canadensis Moench* revealed an lncRNA-gene interactive network, which consisted of four lncRNAs and six genes, indicating that lncRNAs modulate heat shock protein (*HSP*) family genes to deal with heat stress [21]. Ye et al. reported that 30 *Populus trichocarpa* lncRNAs regulated salt response genes simultaneously in cis- and trans-styles, and verified the targeted interaction between *Ptlinc-NAC72* and *PtNAC72.A/B* in dual-luciferase report experiments. *Ptlinc-NAC72* directly upregulates the expression of *PtNAC72.A/B*, suggesting that lncRNAs come into play in the upregulation of the *P. trichocarpa* response to salt stress [22]. In addition, 54 differentially expressed lncRNAs were screened for the RNA sequencing analysis of *P. euphratica*, due to the different growth locations of lanceolate leaves on the bottom and broad-ovate leaves on top. Differentially expressed lncRNAs involved in photoadaptation, protein repair, the stress response, and the growth and developmental pathways interact with mRNAs in response to the microenvironment of *P. euphratica* leaves [23]. Zhou et al. deeply analyzed the regulatory mechanisms of lncRNAs in the root system of *Populus × canescens* saplings under different nitrogen fertilization treatments, and constructed the lncRNA-miRNA-mRNA regulatory networks, which provide new ideas for the study of the regulatory mechanisms of woody plants in response to different nitrogen forms [24]. All of these suggest that the roles of lncRNAs in plants should not be underestimated. So what about the role of lncRNA in plant responses to blue light? This has also aroused a lot of interest. In their study on *Arabidopsis thaliana*’s response to blue light, Sun et al. constructed a ceRNA regulatory network on lncRNA. The research on this regulatory mechanism, BLIL1-miR167-ARF6/8, revealed that the lncRNA BLIL1 plays a role in blue-light-induced photomorphogenesis and responses to mannitol stress in plants [25].

In this study, we analyzed the differentially expressed lncRNAs in *741* poplar seedings treated with blue light and white light using high-throughput deep transcriptome sequencing technology. The target genes and interactions between lncRNAs and miRNAs were explored, and an lncRNA-miRNA-mRNA ceRNA regulatory network was constructed to identify the role of lncRNAs in regulating gene expression; the expression level was then verified using qRT-PCR. This study reveals the regulatory roles of lncRNAs induced by LED blue light during plant growth, thereby providing new insights into light-induced molecular mechanisms and a theoretical basis for lncRNA participation in plant growth and development.

## 2. Results

### 2.1. Analysis of mRNA and lncRNA Expression under Different Light Types

**Figure 1:**
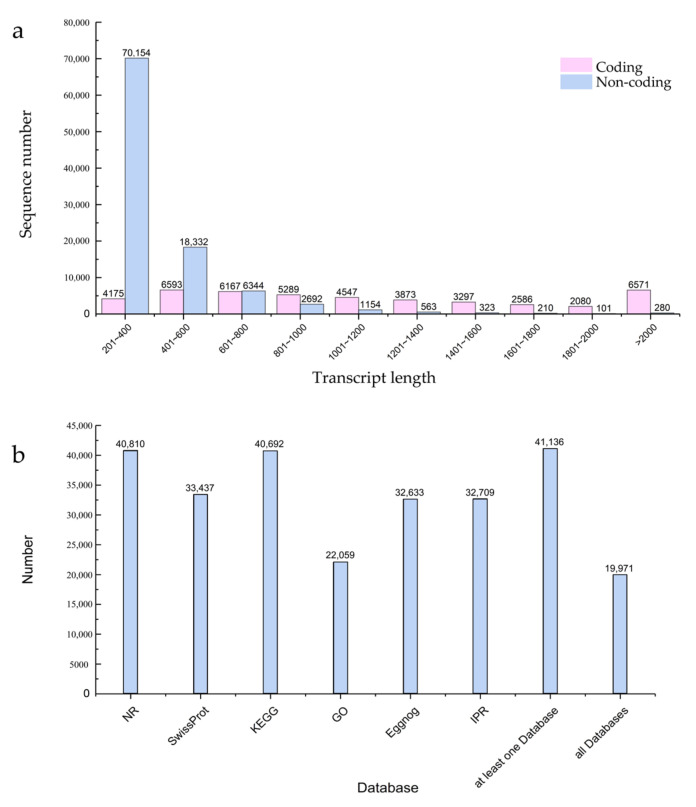
Global data analysis of mRNA and lncRNA expression under different light types in *741* poplar. (**a**) Two types of transcript length distribution plots; (**b**) distribution graph of different databases used for predicting coded transcripts.

Transcriptome sequencing was performed on seeding leaves from 741 poplar after 24 h of blue and white light irradiation. Three biological replicates were used to produce six strand-specific libraries. As shown in Table 1, each library generated over 37,000,000 reads and 5.17 G of bases. The GC content was about 53%, and the Q20 proportion of all replicates exceeded 98.55%, indicating a high reliability of the RNA sequencing data. The sequenced clean reads were mapped with reference genomes to obtain reliable transcripts. The length distribution of the coding transcripts and non-coding transcripts is shown in Figure 1a, with a large number of non-coding transcripts ranging in length from 201 to 400 bp. In addition, the predicted coding transcripts were functionally annotated by searching the NR, Swissprot, KEGG, Eggnog, IPR, and GO databases, as shown in Figure 1b.

### 2.2. Identification and Analysis of Differentially Expressed lncRNAs

**Figure 2:**
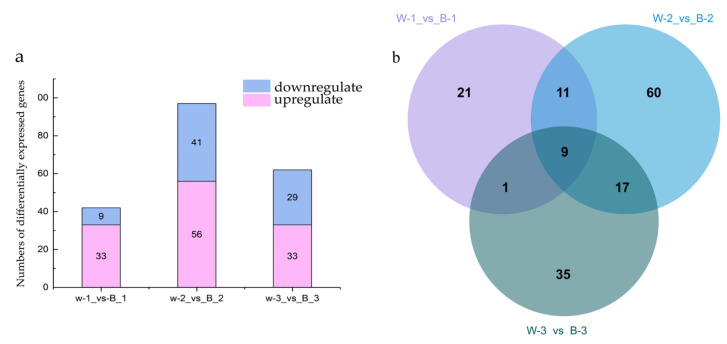
Identification of differentially expressed lncRNAs under blue-white light. (**a**) Numbers of differentially expressed lncRNAs; (**b**) Venn diagram of differentially expressed lncRNAs.

Differentially expressed genes (DEGs) were identified using the R software edgeR package (R Foundation for Statistical Computing, Vienna, Austria), and lncRNA expression levels in poplar seedings exposed to blue and white light for 24 h were compared. The DEGs were genes for which |logFC| > 1 and *p* < 0.05. According to the logFC value statistics, the numbers of upregulated and downregulated differentially expressed lncRNAs obtained in the three groups under the control of blue-white light were 42, 97, and 62, respectively (Figure 2a). In the first group, 33 lncRNAs were upregulated and 9 were downregulated. In the second group, 56 lncRNAs were upregulated and 41 were downregulated; in the third group, 33 lncRNAs were upregulated and 29 were downregulated. The screening shared differentially expressed lncRNAs by making Venn diagrams, as shown in Figure 2b. Nine differentially expressed lncRNAs were obtained. Details are shown in Table 2. mRNA_GeneName represents the gene name of the mRNA where the lncRNA is located. mRNA_region indicates the position of the lncRNA on the corresponding mRNA. Sense strand refers to the strand that is in the same direction as the mRNA strand, while antisense strand refers to the strand that is in the opposite direction to the mRNA strand. UP indicates that the expression was up-regulated after blue light induction, and DOWN indicates that the expression was down-regulated.

### 2.3. GO and KEGG Analyses of the Differentially Expressed lncRNAs Target Genes

**Figure 3:**
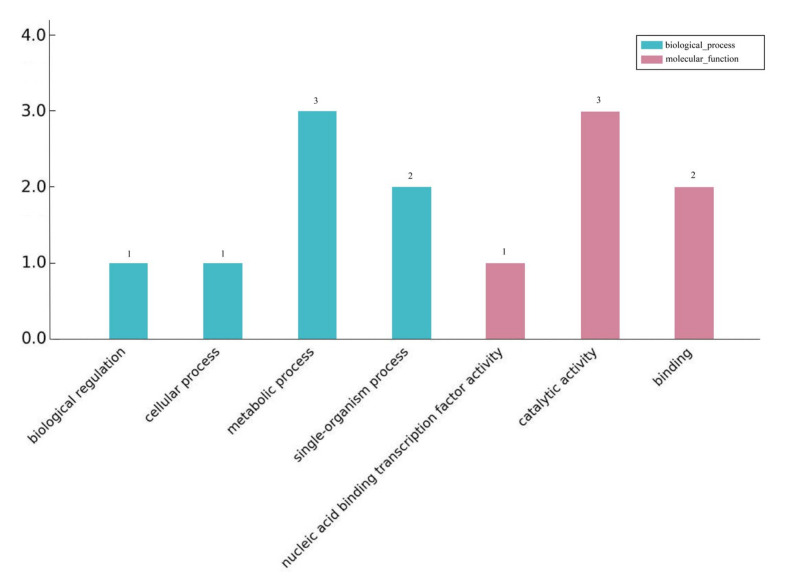
GO annotation of differentially expressed lncRNAs target genes.

The differentially expressed lncRNAs target genes were classified and described according to biological processes, cell composition, and molecular functions. Figure 3 shows the GO classification results of the differentially expressed lncRNAs target genes enriched for biological processes and molecular functions after 24 h of blue and white light. During the biological process, seven genes were enriched. Metabolic processes and single biological processes accounted for 42% and 28% of the total, respectively. Six genes were enriched in molecular functions, of which three were catalytically active. In the KEGG pathway enrichment analysis of the target genes, only one target gene was found to be enriched in the biological synthesis of flavonoids, which are involved in light and biological processes.

### 2.4. Target Gene Prediction Based on Differentially Expressed lncRNAs

Target genes were predicted on the basis of screened lncRNAs. Only three of the nine differentially expressed lncRNAs were predicted to have target genes, and their specific information is shown in Table 3. The target gene functionally annotated by *MSTRG.20734.2* was predicted to be a protein; the target gene was functionally annotated as calcium-modulated binding protein in *MSTRG.7072.1*. The two target gene functions predicted by MSTRG.9936.2 were not functionally annotated, but the proteins that interacted with them were chlorophyll A/B binding proteins, belonging to the Lhca3 and Lhca6 families, respectively.

Using the psRNATarget website to predict the target sites of the differentially expressed lncRNAs, six lncRNAs were found to be the targets of twelve miRNAs in six miRNA families (Table 4).

### 2.5. Verification of Differentially Expressed lncRNAs

**Figure 4:**
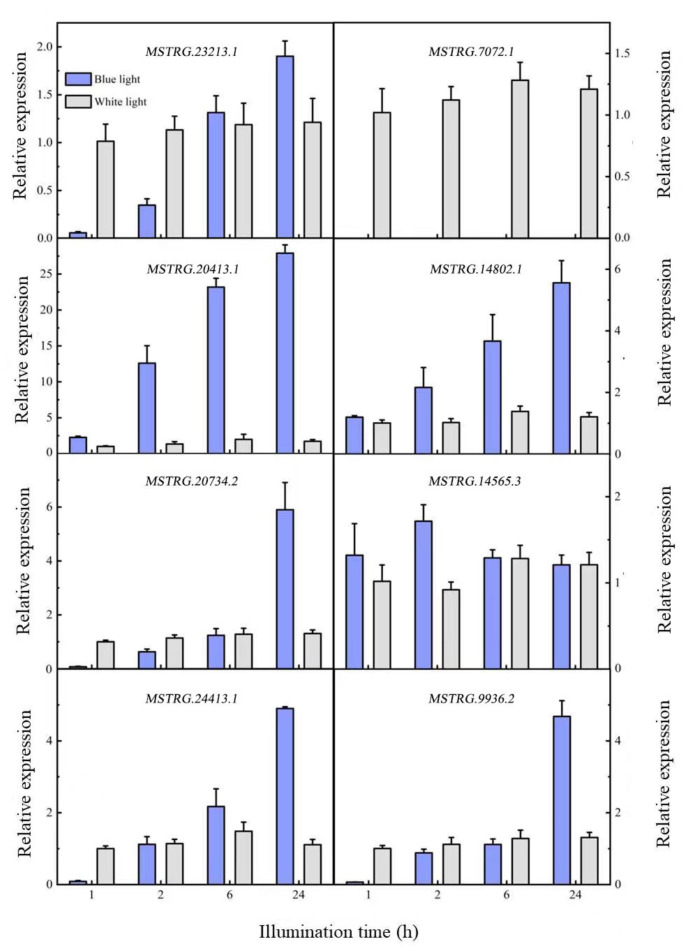
Differential lncRNA expression patterns.

Nine differentially expressed lncRNAs were screened in *741* poplar after being induced by blue light, of which seven were upregulated and two were downregulated. To verify the differential expression of lncRNAs in response to blue light, eight lncRNAs were selected and quantified using qRT-PCR (Figure 4). The expression levels of the eight lncRNAs tended to stabilize within 24 h after white light irradiation, while four expression modes were detected within 24 h of blue light irradiation. In the first mode, lncRNA expression decreased after it was induced by blue light, and then gradually increased with time; the expression exceeded the white light level of the control after 2 and 6 h. *MSTRG.23213.1*, *MSTRG.20734.2*, *MSTRG.24413.1*, and *MATRG.9936.2* had similar expression patterns. In the second mode, the expression level of lncRNA after induction by blue light was similar to that of the white light control, but expression levels then gradually increased, including those of *MSTGR.20413.1* and *MSTRG.14802.1*. In the third mode, lncRNA expression first increased and then decreased after being induced by blue light; the lncRNA expression was similar to that under white light after 24 h, including that of *MSTRG.14565.3*. The last mode was characterized by a downregulated lncRNA expression; the expression suddenly decreased, and no expression was detected after the blue light treatment, including of *MSTGR.7072.1*. In summary, seven of the eight differentially expressed lncRNAs were upregulated, and one was downregulated. This is similar to the transcriptome sequencing results.

### 2.6. Verification of Differentially Expressed lncRNAs and Target Gene Expression

**Figure 5:**
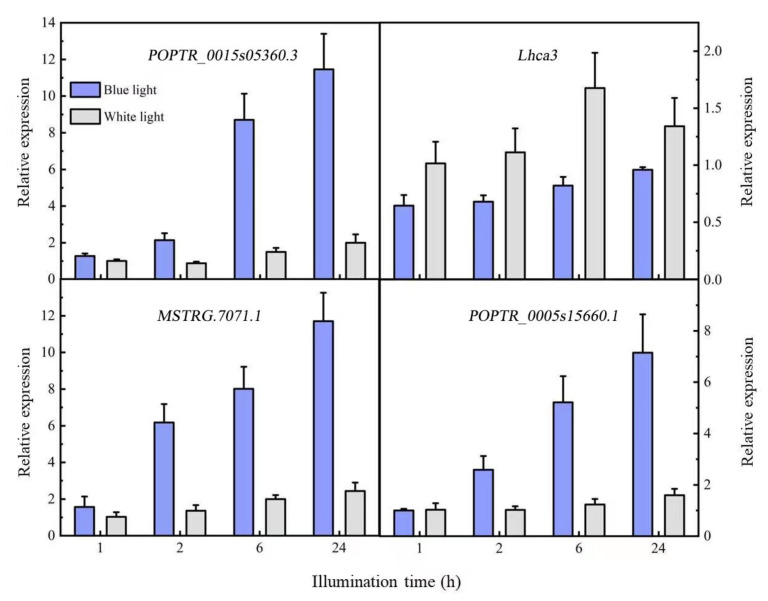
Differentially expressed lncRNA target gene expression patterns.

The predicted target genes were verified with qRT-PCR (Figure 5). The target gene of *MSTRG.7072.1* was *MSTRG.7071.1*, which was functionally annotated as a calcium-modulated binding protein upregulated by blue light; its expression gradually increased with time. The expression pattern was the opposite of that of the corresponding lncRNA. Although the target genes *POPTR_0015s05360.3* and *POPTR_0015s05360.4* of *MSTRG.9936.2* had no functional annotations, their interacting proteins were photosynthesis-related chlorophyll A/B binding proteins (*Lhca3* and *Lhca6*). The qRT-PCR of *POPTR_0015s05360.3* and *Lhca3* showed that the target gene *POPTR_0015s05360.3* was upregulated, while *Lhca3* was downregulated and the lncRNA *MSTRG.9936.2* was upregulated. We speculated that the target gene and interacting protein were negatively regulated, while the lncRNAs and target gene were positively regulated. *POPTR_0005s15660.1* expression increased gradually after induction by blue light, consistent with the expression trend of the lncRNA *MSTRG.20734.2*.

### 2.7. lncRNA-miRNA-mRNA Expression Pattern Analysis

**Figure 6:**
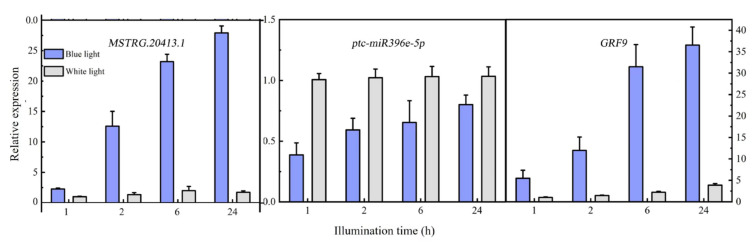
Expression pattern analysis of the lncRNA *MSTRG.20413.1*, *ptc-miR396e-5p* and *GRF9*.

**Figure 7:**
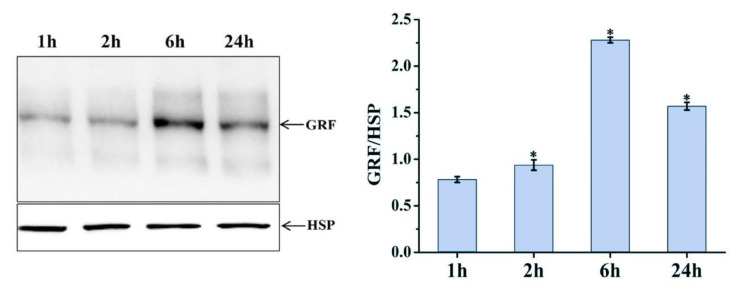
Western blot to detect GRF protein expression. * 0.01 < *p* ≤ 0.05.

Differentially expressed *MSTRG.20413.1* has two target sites in the ptc-miR396 family (*ptc-miR396c* and *ptc-miR396e-5p*), according to the psRNATarget website. These target sites have been used as miRNA target sites, and miRNA regulates the expression of the target genes. For *ptc-miR396c* and *ptc-miR396e-5p*, target gene prediction was also used: PsRNATargrt. They predicted a total of 24 target genes (mostly GRF growth regulators). The lncRNA *MSTRG.20413.1*-*ptc-miR396e-5p*-*GRF9* regulatory network was constructed for expression pattern analysis. Expression patterns at different time points were analyzed with qRT-PCR. Figure 6 shows the expression patterns at 1, 2, 6, and 24 h after blue light irradiation; *ptc-miR396e-5p* expression was downregulated compared with white light, and the expression did not increase significantly over time. The expressions of lncRNA *MSTRG.20413.3* and the *GRF9* were upregulated compared with white light, and the expression increased gradually over time; the expression level tended to be stable under white light. GRF expression levels were detected in leaf whole proteins extracted from leaves exposed to blue light for 1, 2, 6, and 24 h by using Western blot. Figure 7 shows that there was no significant change in the expression of GRF9 protein before being induced by blue light for 6 h. With the prolonged irradiation time, its expression increased significantly after 6h of blue light induction, which was consistent with the results of real-time fluorescence quantification. Thus, the expression of *ptc-miR396e-5p* was downregulated after being induced by blue light, while *MSTRG.20413.1* and *GRF9* were upregulated as targets; that is, lncRNA interacted with miRNA as an miRNA target to regulate gene expression and affect plant growth and development.

## 3. Discussion

*Populus* is widely used to establish fast-growing timber forests [26]. Light is indispensable for plants, which accept light for photosynthesis to carry out growth and development. The regulation of gene expression has been reported to have great impact on the growth and development of plants, as well as on environmental adaptation [27,28]. The expression patterns and regulatory functions of lncRNAs have received attention, and studies have shown that lncRNAs adjust the delivery of targets genes in response to variation in the external environment [29,30]. However, how lncRNAs participate in the regulation of poplar growth and development in response to changes in light conditions has not been reported. In this study, *741* poplar was treated with blue and white light as a control, and lncRNAs showing differential expression in response to blue light irradiation were identified by transcriptome sequencing to shed light on the regulatory mechanisms of lncRNA. LncRNAs act on cis- or trans- to regulate gene expression at the transcriptional, epigenetic modification, or post-transcriptional level [22]. In this study, *741* poplar was irradiated with blue and white light for 24 h, and 16,692 non-coding lncRNAs were identified using transcriptome sequencing; nine differentially expressed lncRNAs were screened out.

LncRNAs function primarily by regulating other RNAs, such as miRNAs and mRNAs. In this study, only three of the nine differentially expressed lncRNAs predicted a target mRNA. One target mRNA was functionally annotated as a calcium-modulated binding protein, and the interactive protein of the two target mRNAs was the chlorophyll A/B binding protein. LncRNAs not only positively regulate target genes, but also “reverse regulate” target genes. Moreover, one lncRNA can regulate one or more target genes [31]. *MSTRG.7071.1*, the *MSTRG.7072.1* target gene, is an important Ca^2+^ binding protein and sensor that regulates downstream target regulatory proteins, and plays a vital role in cell signal transduction, plant growth and development, and the stress response [32]. Studying calcium-modulated binding proteins will shed light on the calmodulin-mediated signal transduction pathways, and could aid in the discovery of specific regulatory processes mediated by lncRNAs. Although the two target genes predicted by the *MSTRG.9936.2* lncRNA do not have biologically annotated functions, their interactive proteins are chlorophyll A/B binding proteins. These pigment–protein complexes are found in photosystem II (PSII), where light energy is absorbed and transmitted in the thylakoid membranes. Light energy is rapidly transferred to reaction centers in photosystem I (PSI) and PSII, and converted into chemical energy; this promotes the photosynthetic reaction [33]. These proteins are involved in processes such as photosynthesis, photoprotection, and adaptation to various environments. We found that the expression of *MSTRG.20734.2* lncRNA was consistent with that of the target gene *MSTRG.7071.1*. Moreover, the expression was upregulated after exposure to blue light; that is, lncRNA positively regulated the expression of the target gene.

Many studies have shown that lncRNAs form miRNA precursors or serve as target miRNA sites through intracellular cleavage [34]. Wang et al. identified 239 differentially expressed lncRNAs in tomatoes involved in the cold damage response, among which 186 competed to bind to 45 miRNAs. Thus, lncRNA, as the miRNA binding site, participated in various metabolic processes, such as redox reactions, cell wall degradation, and cold/HSP formation in plants [35]. A growing body of research suggests interactions between lncRNAs and miRNAs. Fan et al. identified and verified seven pairs of drought-responsive lncRNA-miRNAs in maize, and constructed an lncRNA-miRNA-mRNA regulatory network [36]. Xu et al. constructed a regulatory network for *Qiongtaoensis Populus* seedlings composed of *miR144d*, *miR482a*, *miR530a*, *lncHSP18.2*, *HSP18.1*, *HSP18.2*, lncRNA, miRNA, and mRNA in response to high-temperature stress (40 °C) [37]. In the present study, the differentially expressed *MSTRG.20413.1* had two ptc-miR396 family target sites (*ptc-miR396c* and *ptc-miR396e-5p*), which were used to construct the lncRNA *MSTRG.20413.1-ptc-miR396e-5p-GRF9* regulatory network. *ptc-miR396e-5p* was downregulated after blue light exposure, while lncRNA *MSTRG.20413.1* and *GRF9* were upregulated. This explains the increase in lncRNA expression under certain stimuli and signals: miRNA has a higher probability of binding to lncRNA, such that the target genes downstream of miRNA are no longer inhibited by miRNA. The GRF family is a family of conserved transcription factors in plants that mediate protein–protein and protein–DNA interactions [38]. GRFs in many plants carry the *miR396* target site regulated by the post-transcriptional cleavage of *miR396* [39]. Some studies have reported that the miR396-GRF pathway regulates the growth and development of various organs through cell proliferation and growth, such as leaf expansion, stem and root elongation, and seed and flower formation [40,41]. Zhang et al. identified the miR396 gene family in cotton and determined that the *miR396b-GRF5* regulatory network has a key role in fiber development [42]. This result further illustrates that lncRNAs that respond to light regulate the expression of target genes by interacting with other miRNAs, thereby affecting plants, their growth, and their intrinsic molecular regulatory mechanisms.

## 4. Materials and Methods

### 4.1. Plant Materials

The test material was laboratory bred *741* poplar, which was a hybrid bred by [*P. alba* × (*P. davidiana* + *P. simonii*)] as the female parent and *P. tomentosa* as the male parent. A total of *741* poplar seedings with strong and uniform growth were transplanted in a pot and incubated in an artificial climate chamber under white light for 14 h·d^−1^, with daytime and nighttime temperatures of 27–30 °C and 20–22 °C, respectively, and relative humidity = 75%. When the seedlings were about 10 cm long, they were treated with the LED blue (450 nm) group and white light control group for 24 h, and the light intensity was 16 μmol/(m^2^·s). Each group contained three biological replicates: B-1–B-3 and W-1–W-3. The third functional leaves of the plants were placed in tin foil after 0, 1, 6, and 24 h of treatment and quick-frozen in liquid nitrogen for subsequent transcriptome sequencing and qRT-PCR experiments.

### 4.2. cDNA Library Construction, Sequencing, and Transcript Assembly

The RNA of *741* poplar irradiated with blue and white light for 0 h, 1 h, 6 h, and 24 h were extracted. The method followed was that detailed in the polysaccharide polyphenol plant total RNA extraction kit (TIANGEN Biotech, Co., Ltd., Beijing, China). The quality and concentration of extracted RNA were checked using a NanoDrop ND-1000 spectrophotometer (LabTech, Holliston, MA, USA). Extracted RNA was accurately assayed using an Agilent 2100 (Agilent Technologies, Santa Clara, CA, USA), and RIN (RNA integrity number) > 7 for each sample, and the total RNA in the sample was treated with DNase I. The *741* poplar mRNA was enriched with magnetic beads containing oligo (dT), and the mRNA was cleaved into short fragments by adding a fragmentation buffer. The mRNA was used as a template to synthesize cDNA and purified using a DNA purification kit. The purified cDNA was terminal-repaired, poly-A tail was added, and the sequencing joint was connected. AMPure XP beads were used to select the size of fragments. The 300 bp size DNA fragment was recovered with electrophoresis, and the sequencing sample was enriched using PCR amplification. High-throughput sequencing was performed for each sample, using the Illumina HiSeq 2000 sequencing platform. The original data were filtered out using Cutadapt software and the quality of the clean data thus obtained was evaluated. Clean reads were mapped to reference genomes using Hisat2 2.2.0 [43] software. The StringTie software could accurately assemble transcripts and perform transcript quantification. Using StringTie v2.1.4 software, based on the alignment results to the genome, reads were assembled into transcripts and quantified [44].

### 4.3. Identification of lncRNAs

Transcripts < 200 bp in length with mRNA overlap were filtered out. The transcript encoding potential was predicted using Coding Potential Calculator (CPC) v 2.0 software [45] and the Encode-Noncoding Tool (CNCI) [46], and transcripts with a CPC score < −1 and CNCI score <0 were removed. The transcripts were filtered using the default settings of CPAT [47] and the Pfam database, and an E value of <0.001; the remaining transcripts were considered lncRNAs.

### 4.4. Differential lncRNA Expression Analysis

The number of fragments per kilobase per million was calculated using StringTie v2.1.4 software to analyze gene expression levels. Differentially expressed lncRNAs, whose |logFC| > 1 and *p* < 0.05, were identified using edgeR [48]. RNAs modulating the same function are often co-expressed, and by analyzing RNAs co-expressed with differentially expressed lncRNAs, the potential function of lncRNAs can be reflected to some extent. The expressional correlation between differentially expressed lncRNAs and mRNAs were calculated and screened for *p*-value < 1 × 10^−5^ [49]. GO and KEGG significance enrichment analysis of differentially expressed lncRNA target genes was based on co-expression results.

### 4.5. Differentially Expressed lncRNAs as miRNA Targets: Prediction of Their Target Genes

Antisense analysis was also used in the prediction of LncRNA target genes. Because lncRNAs regulate several post-transcriptional processes, they are frequently connected with base complementary pairing. Target gene predictions for the antisense analysis of differentially expressed lncRNAs using the RNAplex (TBI—Theoretical Biochemistry Group—Software (univie.ac.at)) algorithm and their potential biological functions were analyzed. The psRNATarget (psRNATarget: A Plant Small RNA Target Analysis Server (2017 Update) (zhaolab.org)) website was used to predict the differentially expressed lncRNAs screened as target sites for miRNAs, and the prediction criterion was ≤3.

### 4.6. qRT-PCR Validation of Differentially Expressed lncRNAs and Target Genes

Eight lncRNAs and four target genes were selected to verify the reliability of high-throughput RNA-sequencing using qRT-PCR, as well as the expression patterns of the target genes under blue and white light irradiation. Primer 6.0 software was used to design primers. The primer information is shown in Tables S1 and S2. RNA was extracted from *741* poplar leaves irradiated with blue and white light for 0, 1, 6, and 24 h. RNA was extracted from 741 poplar leaves treated with blue and white light at 0 h, 1 h, 6 h, and 24 h. The lnRcute lncRNA First-Strand cDNA Kit (TIANGEN Biotech, Co., Ltd., Beijing, China) was used for reverse transcription to synthesize cDNA. The InRcute (SYBR Green) IncRNA qPCR Kit (TIANGEN Biotech, Co., Ltd., Beijing, China) was then employed for real-time fluorescence quantitative PCR. The reaction system consisted of 12.5 μL of 2×lnR lncRNA PreMix, 0.625 μL each of the forward and reverse primers, 1 μL of cDNA template, and 10.25 μL of RNase-Free ddH_2_O. The reaction steps were as follows: initial denaturation at 95 °C for 3 min, followed by 40 cycles of denaturation at 95 °C for 5 s, annealing at 60 °C for 10 s, and extension at 72 °C for 15 s. The 18s RNA was selected as an internal control. The Stratagene Mx3005P Real-Time PCR instrument (Agilent Technologies) was used for qRT-PCR. The relative expression level of the gene was calculated using the 2^−ΔΔCT^ method.

RNA was reverse-transcribed into cDNA for the fluorescent quantification of target genes using the FastKing gDNA Dispelling RT SuperMix. Amplification was performed using the SYBR Premix Ex Taq II kit (Takara Biotech, Dalian, China). The reaction system included 10 μL of mix, 0.5 μL each of forward and reverse primers, 2 μL of cDNA template, and 7 μL of ddH_2_O. The reaction program consisted of an initial denaturation at 95 °C for 5 min, followed by 40 cycles of denaturation at 95 °C for 30 s, annealing at 56 °C for 30 s, and extension at 72 °C for 30 s. The 18 s RNA was selected as an internal control.

### 4.7. LncRNA-miRNA-mRNA Expression Pattern Validation

We used qRT-PCR to analyze the expression profiles of *MSTRG.20413.1*, *ptc-miR396e-5p*, and the corresponding target gene (*GRF9*) within 24 h of blue and white light irradiation. Primers were designed using Primer 6.0 software, as shown in Table S3. RNA was reverse-transcribed into cDNA using the miRcute Plus miRNA First-Strand cDNA Kit (TIANGEN Biotech, Co., Ltd., Beijing, China) to generate a fluorescent quantification template for ptc-miR396e-5p. Amplification was performed using the miRcute Plus miRNA qPCR (SYBR Green) Kit (TIANGEN Biotech, Co., Ltd., Beijing, China). The reaction system included 10 μL of 2×miRcute Plus miRNA PreMix (SYBR&ROX), 0.4 μL each of forward and reverse primers, 1 μL of miRNA first-strand cDNA, and 8.2 μL of ddH_2_O. The amplification program consisted of an initial denaturation at 95 °C for 15 min, followed by 43 cycles of denaturation at 94 °C for 20 s, and annealing at 60 °C for 34 s. U6 was selected as the reference gene. The reaction system and program for GRF9 were set up and performed in the same manner as described in Section 4.6 for the fluorescent quantification of target genes. The Stratagene Mx3005P Real-Time PCR instrument (Agilent Technologies) was used for qRT-PCR. The relative expression level of the gene was calculated using the 2^−ΔΔCT^ method.

### 4.8. Analysis of Protein Expression in Poplar Leaves Using Western Blotting

To further validate the expression levels of miRNA target genes induced by blue light, total leaf proteins were extracted from leaves treated with blue light for 1 h, 2 h, 6 h, and 24 h. Western blotting was performed to analyze the expression of GRF9. The extracted poplar protein samples were mixed with sample loading buffer and denatured at 100 °C for 5 min. An SDS-PAGE gel was prepared, and the samples were loaded and subjected to electrophoresis at 160 V for 60 min. A PVDF membrane was prepared, and after electrophoresis the proteins were transferred onto the membrane at a voltage of 100 V for 60 min. After transfer, the membrane was blocked for 1 h. Following blocking, the PVDF membrane was incubated with a primary antibody specific to GRF9 diluted in a 5% non-fat milk TTBS solution at room temperature for 3 h. After the primary antibody incubation, the membrane was washed three times for 5 min each with TTBS solution. Then, the membrane was incubated with a secondary antibody diluted in a 5% non-fat milk solution at room temperature for 1.5 h. After the secondary antibody incubation, the membrane was washed three times for 5 min each with TTBS solution. Subsequently, 10 mL of BCIP/NBT substrate reaction solution was added to the membrane for color development in the dark, and the reaction was stopped with ddH_2_O. The image was captured using a chemiluminescence imaging system. After stripping the target protein antibody, the membrane was incubated with an HSP [50,51] reference antibody, and the detection results served as the internal control for equal loading.

## 5. Conclusions

In this study, high-throughput transcriptome sequencing was conducted after the blue and white light treatment of *741* poplar to identify differentially expressed lncRNAs and explore the mechanisms through which lncRNA regulates the growth and development of *741* poplar in response to blue light. Our results show that the target genes of three differently expressed lncRNAs were functionally annotated, and the functional annotations provided an important reference for the functional study of lncRNAs. Six lncRNAs served as twelve miRNA target sites for six miRNA families. In the constructed lncRNA *MSTRG.20413.1-ptc-miR396e-5p-GRF9* regulatory network, *ptc-miR396e-5p* was downregulated after blue light exposure, while lncRNA *MSTRG.20413.1* (as the target) and *GRF9* were upregulated, further indicating that lncRNAs interact with miRNAs to regulate gene expression and affect plant growth and development. These findings provide a theoretical basis for a functional study of lncRNAs in other plants, and also provide new insights to aid further research on the molecular mechanisms of light induction.

## Figures and Tables

**Table 1:** Transcriptome data of the six strand-specific libraries under blue-white light.

	Read	Base	Q 20%	Q 30%	GC Content%
WHT-1	46155433	6.9G	98.57%	95.68%	53%
WHT-2	37327425	5.6G	98.61%	95.74%	53%
WHT-3	42099884	6.3G	98.55%	95.62%	53%
LBL-1	34499736	5.2G	98.58%	95.65%	52%
LBL-2	51038681	7.6G	98.57%	95.67%	53%
LBL-3	40665290	6.1G	98.56%	95.68%	52%

**Table 2:** Information about the differentially expressed lncRNAs.

No.	lnc Gene	mRNA_GeneName	mRNA_Region	Strand_Status	Up or Down
1	MSTRG.23213.1	POPTRDRAFT_0006s24570	cds	antisense	up
2	MSTRG.20734.2	POPTRDRAFT_580797	cds	antisense	
	MSTRG.20734.2	POPTRDRAFT_580797	cds	antisense	up
	MSTRG.20734.2	POPTRDRAFT_580797	utr5	antisense	
3	MSTRG.24413.1	POPTRDRAFT_1083565	downstream2K	sense	
	MSTRG.24413.1	POPTRDRAFT_1083561	upstream2K	sense	up
	MSTRG.24413.1	POPTRDRAFT_1083565	utr3	sense	
	MSTRG.24413.1	POPTRDRAFT_1083565	cds	sense	
	MSTRG.24413.1	POPTRDRAFT_1083565	utr5	sense	
4	MSTRG.7072.1	MSTRG.7071	exonic	antisense	down
	MSTRG.7072.1	MSTRG.7071	intronic	antisense	
5	MSTRG.14802.1	POPTRDRAFT_550790	cds	antisense	
	MSTRG.14802.1	MSTRG.14809	intronic	antisense	up
	MSTRG.14802.1	POPTRDRAFT_753933	intronic	antisense	
6	MSTRG.14565.3	POPTRDRAFT_580392	exonic	sense	
	MSTRG.14565.3	POPTRDRAFT_580392	intronic	sense	up
	MSTRG.14565.3	POPTRDRAFT_580392	downstream2K	sense	
7	MSTRG.9936.2	MSTRG.9934	downstream2K	sense	up
	MSTRG.9936.2	POPTRDRAFT_580622	cds	antisense	
	MSTRG.9936.2	MSTRG.9934	downstream2K	sense	
	MSTRG.9936.2	POPTRDRAFT_580622	intronic	antisense	
8	MSTRG.25033.1				down
9	MSTRG.20413.1				up

**Table 3:** Target gene prediction information for the differentially expressed lncRNAs.

lnc RNA	Target Gene	Function Notes
MSTRG.20734.2	POPTR_0005s15660.1	Predictive protein
MSTRG.7072.1	MSTRG.7071.1	Calmodulin binding protein
MSTRG.9936.2	POPTR_0015s05360.3	Interaction protein: chlorophyll A-B binding protein (Lhca3) Interaction protein: chlorophyll A-B binding protein (Lhca6)
	POPTR_0015s05360.4	

**Table 4:** Differentially expressed lncRNAs as predictors of miRNA target sites.

miRNA Acc.	Target Acc. LncRNA	Alignment													
ptc-miR156h	LOC7487123	miRNA	21	C A	C G	A G	A G	A U	A G	A A	G A	C A	G U	U	1
				: :	:	:	: :	: :	: :	: :	. :	: :	: :		
		Target	762	G U	C C	U G	U C	U A	U C	U U	U U	G U	C A	U	782
ptc-miR166c	LOC112328927	miRNA	21	C C	C C	U U	A C	U U	C G	G A	C C	A G	G C	U	1
				: :	: :	: :	: :	: :	: :	: :	: :	: :	: :	:	
		Target	301	G G	G G	A A	U G	A A	G C	C U	G G	U C	C G	A	321
ptc-miR166e		miRNA	21	C C	C C	U U	A C	U U	C G	G A	C C	A G	G C	U	1
				: :	: :	: :	: :	: :	: :	: :	: :	: :	: :	:	
		Target	301	G G	G G	A A	U G	A A	G C	C U	G G	U C	C G	A	321
ptc-miR166i		miRNA	21	C C	C C	U U	A C	U U	C G	G A	C C	A G	G C	U	1
				: :	: :	: :	: :	: :	: :	: :	: :	: :	: :	:	
		Target	301	G G	G G	A A	U G	A A	G C	C U	G G	U C	C G	A	321
ptc-miR167b	MSTRG.25033.1	miRNA	21	A U	C U	A G	U A	C G	A C	C G	U C	G A	A G	U	1
				: :	: :	:	: :	: :	:	: :	: :	:	: :	:	
		Target	1654	U A	G A	U --	A U	G C	U U	G C	A G	C G	U C	A	1673
ptc-miR167f-5p		miRNA	21	U U	C U	A G	U A	C G	A C	C G	U C	G A	A G	U	1
				:	: :	:	: :	: :	:	: :	: :	:	: :	:	
		Target	1654	U A	G A	U --	A U	G C	U U	G C	A G	C G	U C	A	1673
ptc-miR319a	LOC112325201	miRNA	20	U U	C U	C G	A G	G G	A A	G U	A G	G U	U U		1
				: :	: :	:	.	: .	: :	: :	: :	:	: .		
		Target	521	G G	G A	U C	A U	C U	U U	C A	G U	C A	A G		540
ptc-miR319d		miRNA	20	U U	C U	C G	A G	G G	A A	G U	A G	G U	U U		1
				: :	: :	:	.	: .	: :	: :	: :	:	: .		
		Target	521	G G	G A	U C	A U	C U	U U	C A	G U	C A	A G		540
ptc-miR396c	MSTRG.20413.1	miRNA	21	U U	C A	A G	U U	C U	U U	C G	A C	A C	C U	U	1
						: :	: :	: :	. .	:	: :	: :	: :	.	
		Target	1159	G A	A A	U C	A A	G A	G G	G A	U G	U G	G A	G	1179
ptc-miR396e-5p		miRNA	21	U U	C A	A G	U U	C U	U U	C G	A C	A C	C U	U	1
						: :	: :	: :	. .	:	: :	: :	: :	.	
		Target	1159	G A	A A	U C	A A	G A	G G	G A	U G	U G	G A	G	1179
ptc-miR403b	LOC7481412	miRNA	21	G C	U C	A A	A C	A C	G C	A C	U U	A G	A U	U	1
				:	. :	: :	: :	: :	:	: :	: :	: :	: :	:	
		Target	278	C A	G G	U U	U G	U G	C A	U G	A A	U C	U A	A	298
ptc-miR403c-3p		miRNA	21	G C	U C	A A	A C	A C	G C	A C	U U	A G	A U	U	1
				:	. :	: :	: :	: :	:	: :	: :	: :	: :	:	
		Target	278	C A	G G	U U	U G	U G	C A	U G	A A	U C	U A	A	298

## Data Availability

The data is contained within the manuscript and Supplementary materials.

## Supplementary Materials

The following supporting information can be downloaded at: https://www.mdpi.com/article/10.3390/plants12173157/s1, Table S1: qRT-PCR primer information for lncRNA; Table S2: qRT-PCR primer information for the lncRNA target genes; Table S3: Primer information for lncRNA *MSTRG.20413.1*, *ptc-miR396e-5p*, and *GRF*.
