# Supplementary material for: Identification of Differentially Expressed lncRNAs in Response to Blue Light and Expression Pattern Analysis of Populus tomentosa Hybrid Poplar 741
Source: Plants (Basel). 2023 Sep 2;12(17):3157. doi: 10.3390/plants12173157 (PMC10490017; doi:10.3390/plants12173157)
Supplement: Supplementary file 1 [file plants-12-03157-s001.zip › plants-2489541-supplementary.pdf]

Supplementary Materials

Table S1. qRT-PCR primer information of lncRNAs.

Primer name	Primer sequence (5'-3')
MSTRG.23213.1	F：TGTCCTTAAGATCTGCGGTA<br>R：GATTGCCTATGTCAAATGCTT
MSTRG.20734.2	F：AGGCGATAAAACTGATGCACT<br>R：CTACACATGTTTGGATGCACT
MSTRG.24413.1	F：AGGCAGAGTTCCATTCCCT<br>R：TACCTTATGAACCAATGCCTT
MSTRG.7072.1	F：ATTACATCAATCCGAAACACT<br>R：CGTACCATTTCTTATCGGTT
MSTRG.20413.1	F：AACAACAACCTTCTAGTGCAT<br>R：TCTAAATCCACCACGACCTCA
MSTRG.14802.1	F：GCCCAACTAATAAACACCCTT<br>R：AAGTACACAGCCTTAGGAGC
MSTRG.14565.3	F：GCTGATTTTCTTTGTGCCAT<br>R：CACGTAACCTCAAGATGCC
MSTRG.9936.2	F：GTGTCTTCTCAATGATGCT<br>R：GTTTCACTTCAAAGGTATGCG
18S RNA	F：TTAACGAGGATCCATTGGAGGGCA<br>R：ACCCAACCCAAAGTCCAACTACGA

Table S2. qRT-PCR primer information of target gene.

Primer name	Primer sequence (5'-3')
MSTRG.7071.1	F：TACTGGCAAACAACTTGAGC<br>R：TACCTCATTAGACCCGCAAC
POPTR_0015s05360.3	F：TGGATTAAAGGCTTCACGAT<br>R：TTTGAAGTGTCTCCGCATC
MSTRG.14809.1	F：ATGGCGTACTACAATTATCTGCT<br>R：ATCATCCACTGCCTTGTCT
Lhca3	F：GCGGACCCTTGTTTAACCC<br>R：ACCTGCACAAAGTAACCCAA
Lhca6	F：CCGCTACTTTCATCTAAGCTTG<br>R：ACGTTCTACTAGCTCCAATTCCC
18S RNA	F：TTAACGAGGATCCATTGGAGGGCA<br>R：ACCCAACCCAAAGTCCAACTACGA

Table S3. Expression pattern validation of qRT-PCR primer information.

Primer name	Primer sequence (5'-3')
MSTRG.20413.1	F：AACAACAACCTTCTAGTGCAT<br>R：TCTAAATCCACCACGACCTCA
ptc-miR396e-5p	CCCTTCCACGGCTTGCTTGAACTT
GRF9	F：AGAAGTATTGTGAACGCCAT<br>R：ATCTGTGAACTTACTTTGGCTA
U6	AACGCTTCACGAATTTGCGT
18S RNA	F：TTAACGAGGATCCATTGGAGGGCA<br>R：ACCCAACCCAAAGTCCAACTACGA
